# Long-Term Outcomes after Stroke in Elderly Patients with Atrial Fibrillation: A Hospital-Based Follow-Up Study in China

**DOI:** 10.3389/fnagi.2016.00056

**Published:** 2016-03-29

**Authors:** Yuguang Zhao, Chunying Zou, Cui Wang, Yongbo Zhang, Shuang Wang

**Affiliations:** ^1^Department of Cell Biology, Harbin Medical UniversityHarbin, China; ^2^Department of Neurology, The First Affiliated Hospital of Jiamusi UniversityHeilongjiang, China; ^3^Department of Community Health, Land Reclamation General HospitalHarbin, China; ^4^Department of Neurology, Capital Medical University Affiliated Friendship HospitalBeijing, China; ^5^Department of Neurology, The Second Affiliated Hospital of Harbin Medical UniversityHarbin, China

**Keywords:** ischemic stroke, atrial fibrillation, outcomes, risk factors, elderly

## Abstract

**Background:** Atrial fibrillation (AF) significantly increases the risk of stroke and disease burden; it is an established predictor of poor outcomes after stroke. However, reported differences in outcomes after stroke among elderly patients between AF and non-AF group are conflicting. We aimed to compare differences in outcomes at 1 year and 3 years after stroke among elderly patients (aged ≥ 75 years old) between AF group and non-AF group.

**Methods:** We recruited 1070 consecutive elderly patients who experienced acute ischemic stroke between January 2008 and December 2013 in Jiamusi University First Hospital, China in this study. Information regarding stroke subtype, severity, risk factors, and outcome (mortality, dependency, and recurrence) at 3 and 12 months after stroke were recorded and assessed between AF group and non-AF group.

**Results:** The prevalence of AF was 16.1% overall, with a similar trend in the prevalence of AF between men and women. The AF group were more likely to experience severe stroke compared to the non-AF group (32.0 vs. 11.9%, respectively; *P* < 0.001). There were no obvious differences between groups regarding the prevalence of hypertension, dyslipidemia, obesity, current smoking, and alcohol drinking but there was a higher prevalence of diabetes in the non-AF group (20.3 vs. 30.1%, *P* = 0.010). Mortality and dependency rates were significantly higher in the AF group than in the non-AF group at 1 year after stroke (29.6 vs. 17.8%, *P* = 0.001 for mortality; and 59.5 vs. 36.1%, *P* = 0.010 for dependency) and 3 years after stroke (46.1 vs. 33.2%, *P* = 0.032 for mortality; and 70.7 vs. 49.2%, *P* = 0.010 for dependency); however, there was no significant between-groups differences in rates of recurrence across the follow-up periods. The results for dependency remained stable after adjustment for sex, stroke severity, and stroke risk factors at 3 years after stroke (OR, 2.26; 95% CI, 1.06–4.81; *P* = 0.034); however, the relationship between AF and mortality and recurrence disappeared after adjusting for these covariates

**Conclusion:** These findings suggest that it is crucial to highlight the treatment of elderly stroke patients with AF in order to reduce poor outcomes among the elderly and to reduce the burden of AF in China.

## Introduction

Stroke was the second most common cause of death and the third most common cause of reduced disability-adjusted life-years (DALYs) worldwide in 2010 (Lozano et al., 2012). Stroke incidence has declined in industrialized countries over the last 30 years, but it has recently become the leading cause of death in rural areas and the third cause of death in urban areas in China (Lloyd-Jones et al., 2009, 2010; Redon et al., 2011; The Ministry of Health of the People's Republic of China, 2011).

Atrial fibrillation (AF) is the most common sustained cardiac arrhythmia. It is an independent risk factor for stroke and is associated with marked morbidity, mortality, and socioeconomic burden (Wolf et al., 1991, 1998; Chugh et al., 2001). It confers a five- to six-fold increased risk for ischemic stroke (Romero and Wolf, 2013). The prevalence of AF increases substantially with advancing age (Kannel et al., 1998). Aging is the most important non-modifiable risk factor for stroke (Murray and Lopez, 1997; Lloyd-Jones et al., 2009). Moreover, older patients reportedly have poor functional outcomes after stroke (Palnum et al., 2008; Saposnik et al., 2008; Fonarow et al., 2010; Soares et al., 2011; Deng et al., 2012). Although there is a low prevalence of AF in China, the disease burden of AF-related stroke is great due to the aging population. It is predicted that the number of people aged >60 years worldwide is estimated to increase to 1.2 billion by 2025, double the number of people who were in this age group in 1995 (Krug et al., 2002). The number of people aged ≥65 years in China was 200 million in 2014 (Peilin et al., 2014), and 3.5% of the population was ≥75 years old in 2013 (National Bureaus of Statistics of the People's Republic of China, 2014). Thus, with the aging of the worldwide population, stroke has become a major public health problem.

Stroke patients with AF have been reported to have higher mortality compared to stroke patients without AF, with rates between 30.5 and 63.0% at 12 months after stroke (Kolominsky-Rabas et al., 2001; Roquer et al., 2003; Banks and Marotta, 2007; Saposnik et al., 2008). In addition, worse neurological function has been reported in stroke patients with AF (Stuart-Shor et al., 2009). However, long-term outcomes after stroke in elderly patients with AF are uncertain in China.

Therefore, in this study, we aimed to compare differences in long-term outcomes, including mortality, dependency, and recurrence rates after stroke, between elderly stroke patients with AF and those without AF.

## Materials and methods

This study used data from a stroke registry in the Department of Neurology, Jiamusi University First Hospital, China; the inclusion criteria for stroke patients have been described in a previous study (Wu et al., 2015). Briefly, we prospectively collected data on the clinical characteristics and outcomes for all ischemic stroke patients who were admitted to the Department of Neurology, Jiamusi University First Hospital, China between January 2008 and December 2013. Stroke events were defined according to the World Health Organization's criteria, and all cases of stroke were confirmed by neuroimaging (1989). Patients with transient ischemic attack were excluded from this study, and all stroke patients aged 75 years and over were included. AF was defined as a history of AF, confirmed by at least one electrocardiogram or the presence of arrhythmia during hospitalization.

The ethics committee of Jiamusi University First Hospital approved the study, and written informed consent was obtained from all patients or their next-of-kin.

Data collection and outcome evaluation were performed by senior neurologists who used standardized variable definitions and scores. Stroke subtypes, which were classified on admission, included total anterior circulation infarcts, partial anterior circulation infarcts, lacunar infarcts, and posterior circulation infarcts, according to Oxfordshire Community Stroke Project criteria (Bamford et al., 1991). Stroke severity was categorized into three groups according to National Institutes of Health Stroke Scale (NIHSS) scores: mild (NIHSS score: ≤ 7), moderate (NIHSS score: 8–16), and severe (NIHSS score: ≥17) (Kim et al., 2010). Receiving oral anticoagulants was defined as receiving oral anticoagulants before stroke onset. General stroke risk factors, including hypertension, diabetes mellitus (DM), and hyperlipidemia, were defined according to self-reported previous medical history, and obesity was defined as a body mass index ≥30 kg/m^2^. The NIHSS score and Barthel Index (BI) were evaluated on admission and at discharge; the modified Rankin Scale (mRS) score was assessed on admission, at discharge, and at 3 and 12 months after stroke.

Outcomes included mortality, dependency, and recurrence rates at 3 and 12 months after stroke. Mortality was defined as all-cause cumulative death at the corresponding follow-up time point. Stroke recurrence was defined as all new-onset vascular events, including stroke, myocardial infarction, and venous thrombosis. Dependency was defined as an mRS score >2 (Banks and Marotta, 2007). Follow-up was implemented according to a predetermined procedure; the same senior neurologist collected data at 1 year and 3 years after stroke. Follow-up occurred for all patients in a face-to-face interview, except for those patients who were re-examined in their local hospitals, who completed follow-up by telephone.

Patients were divided into two groups—those with AF (the AF group) and those without AF (the non-AF group)—and data were compared between the two groups. Continuous variables, including age, NIHSS score, BI, mRS score, total cholesterol (TC), triglycerides (TG), high-density lipoprotein cholesterol (HDL-C), low-density lipoprotein cholesterol (LDL-C), fasting glucose, and glycosylated hemoglobin (HbA1c), were presented as means with standard deviations (or as medians with ranges where appropriate) and were compared between groups using the Student *t*-test or the Mann-Whitney *U* test. Dichotomous variables, including stroke severity, stroke risk factors, and outcomes during follow-up after stroke, were presented as number of cases (percentages); the risk factors were compared between groups with the chi-squared test. The relationship between AF and outcomes was assessed with logistic regression analyses and presented as unadjusted odds ratios (ORs) with 95% confidence intervals (CIs). A multivariate analysis between age and outcomes was carried out with a logistic regression analysis, with covariates of stroke severity, previous medical history, and risk factors, and were presented using adjusted ORs with 95% CIs. All statistical analyses were performed using SPSS version 15.0 (SPSS Inc., Chicago, IL), and a two-tailed *p* < 0.05 indicated statistical significance.

## Results

**Figure 1:**
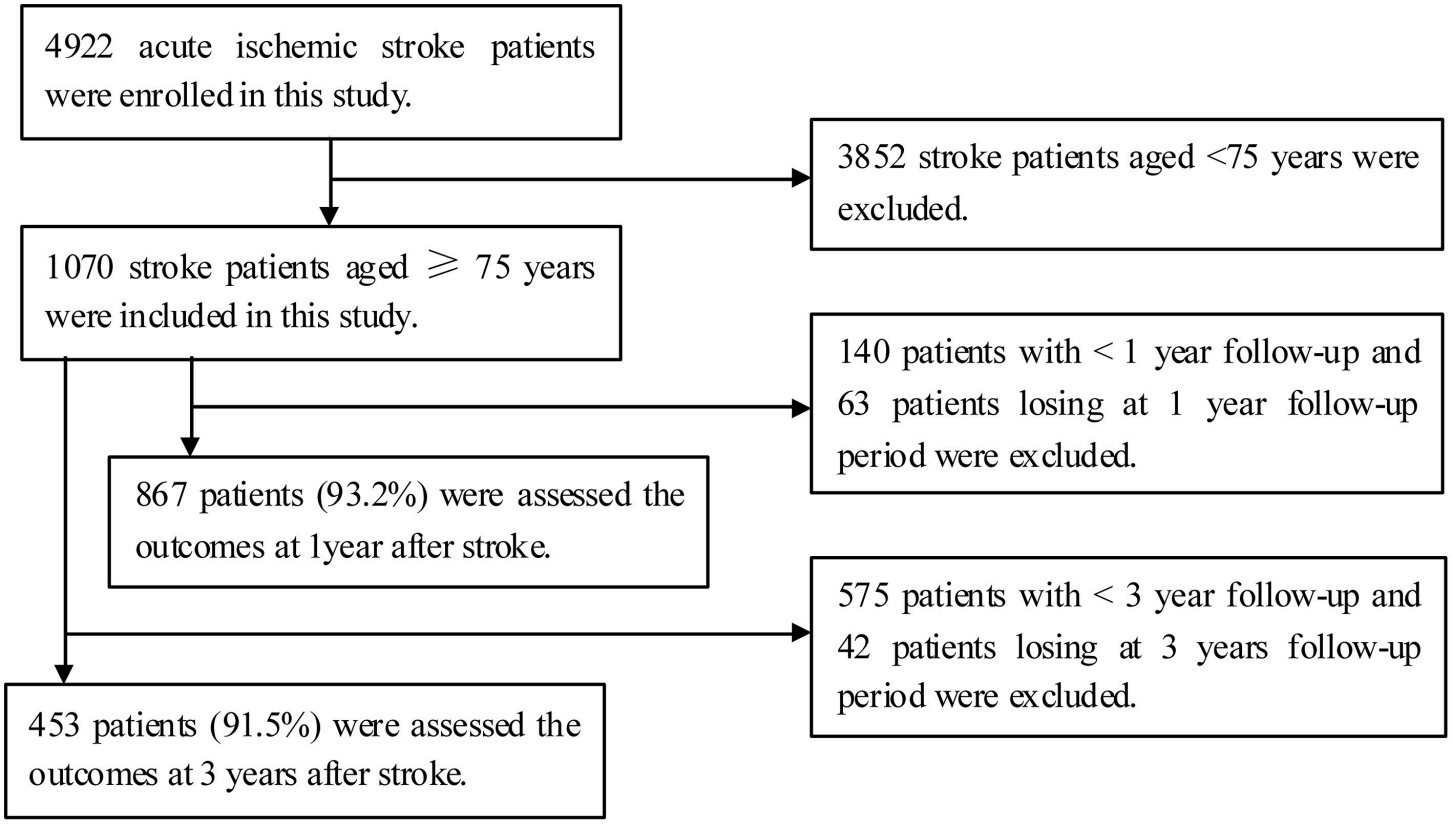
**Flow diagram of participants**.

A total of 4922 patients experienced acute ischemic stroke and were hospitalized during the study period; of these, there were1070 patients aged ≥75 years. Outcomes at 1 year post-stroke were available for 867 elderly stroke patients (93.2%), after excluding 63 patients lost to follow-up, and outcomes at 3 years post-stroke were available for 453 elderly stroke patients (91.5%), after excluding 42 patients lost to follow-up (Figure 1).

Overall, 1070 elderly stroke patients were included in this study (172 in the AF group and 898 in the non-AF group); the prevalence of AF was 16.1% overall; similar for men and women (15.0 vs. 17.5%, *P* = 0.256). The AF group was more likely than the non-AF group to have severe stroke (32.0 vs. 11.9%, *P* < 0.001), and they had worse neurological function (i.e., they had higher mRS scores but lower BI scores). The levels of TC, TG, HDL-C, LDL-C, fasting glucose, and HbA1c were not significantly different between the two groups. There were no obvious differences in the prevalence of hypertension, DM, dyslipidemia, obesity, and current smoking between the two groups, but there was a higher prevalence of diabetes in the non-AF group (Table 1).

**Table 1:** **The clinical features and previous history of diseases in elderly ischemic stroke patients by AF**.

**Characteristics**	**AF group**	**non-AF group**	***P***
Cases, n (%)	172 (16.1)	898 (83.9)	–
TOAST classification, n (%):			<0.001
Atherothrombotic	68 (39.5)	655 (72.9)	
Small artery disease	91 (52.9)	207 (23.1)	
Cardiac embolism	12 (7.0)	21 (2.3)	
Others determined etiology	1 (0.6)	6 (0.7)	
Undetermined etiology	0	9 (1.0)	
OCSP classification, n (%):			
Total anterior circulation infarct	38 (22.1)	55 (6.1)	
Partial anterior circulation infarct	91 (52.9)	514 (57.4)	
Lacunar infarct	6 (3.5)	63 (7.0)	
Posterior circulation infarct	37 (21.5)	264 (29.5)	
Stroke severity, n (%):			<0.001
Mild	70 (40.7)	527 (58.7)	
Moderate	47 (27.3)	264 (29.4)	
Severe	55 (32.0)	107 (11.9)	
Neurological function:			
BI	20 (100)	40 (100)	<0.001
mRS	5 (5)	4 (6)	<0.001
Receiving oral anticoagulants, n (%)	24 (14.0)	0	–
Laboratory examination:			
Total cholesterol	4.78 (1.20)	4.83 (1.09)	0.600
Triglyceride	1.20 (0.99)	1.31 (0.89)	0.156
High density lipoprotein cholesterol	1.16 (0.29)	1.12 (0.61)	0.427
Low density lipoprotein cholesterol	2.95 (0.98)	2.99 (0.88)	0.615
Fasting glucose	6.66 (3.16)	6.49 (2.44)	0.489
Glycosylated hemoglobin	6.27 (1.27)	6.55 (1.25)	0.069
Hypertension	117 (68.0)	663 (73.8)	0.116
Diabetes	35 (20.3)	270 (30.1)	0.010
Dyslipidemias	37 (21.5)	209 (23.3)	0.615
Obesity	23 (13.4)	123 (13.7)	0.909
Current smoking	35 (20.3)	200 (22.3)	0.577
Alcohol consumption	7 (4.1)	71 (7.9)	0.076

Mortality was significantly higher in the AF group than in the non-AF group at 1 year after stroke (29.6 vs. 17.8%, *P* = 0.010) and at 3 years after stroke (46.1 vs. 33.2%, *P* = 0.032), respectively. Similar trends were found for the dependency (49.5 vs. 36.1% at 1 year after stroke and 70.7 vs. 49.2% at 3 years after stroke (all *P* < 0.001). However, there were no significant differences in recurrence rates between the two groups across the follow-up periods. The results for dependency remained stable after adjustment for sex, stroke severity, and stroke risk factors at 3 years after stroke (OR, 2.26; 95% CI, 1.06–4.81; *P* = 0.034); however, the relationship between AF and mortality and recurrence disappeared after adjusting for these covariates (Table 2).

**Table 2:** **The outcomes and OR at 1 year and 3 years after stroke among elderly stroke patients by AF**.

**Outcomes**	**AF group**	**non-AF group**	**Univariate analysis**		**Multivariate analysis**	
			**OR (95%CI)**	***P***	**OR (95%CI)**	***P***
**1 YEAR**						
Mortality	42 (29.6)	129 (17.8)	1.66 (1.23–2.24)	0.001	1.06 (0.79–2.00)	0.335
Dependency	50 (49.5)	216 (36.1)	1.37 (1.10–1.72)	0.010	1.36 (0.86–2.16)	0.187
Recurrence	40 (32.3)	162 (24.1)	1.34 (1.01–1.79)	0.054	1.35 (0.88–2.08)	0.171
**3 YEARS**						
Mortality	35 (46.1)	125 (33.2)	1.39 (1.05–1.84)	0.032	1.28 (0.73–2.23)	0.386
Dependency	29 (70.7)	125 (49.2)	1.44 (1.14–1.82)	0.010	2.26 (1.06–4.81)	0.034
Recurrence	33 (48.5)	158 (45.5)	1.07 (0.81–1.40)	0.650	1.09 (0.63–1.87)	0.767

## Discussion

To our knowledge, this is the first report of the effects of AF on outcomes among elderly stroke patients in China. In this study, we compared the differences in clinical features, conventional stroke risk factors, and outcomes at 1 year and 3 years after stroke among elderly stroke patients with and without AF.

A previous study indicated that, compared to stroke patients without AF, those with AF were less likely to have conventional risk factors for stroke (including hypertension, DM, current smoking, and alcohol consumption Sun et al., 2015), were slightly more likely to experience a recurrent ischemic stroke or intracranial hemorrhage within 2 weeks, and were more than twice as likely to die within 2 weeks to 6 months (Mattle, 2003). Consistent with these studies, there was a higher frequency of severe stroke, and worse neurological function in the AF group than in the non-AF group in this study.

Regarding risk factors for stroke in patients with AF, conclusions are widely divergent due to the different study designs used. The previous study indicated that stroke patients with AF were less likely to have the conventional risk factors for stroke, including hypertension, diabetes mellitus, current smoking, and alcohol consumption habits (Lamassa et al., 2001; Mizrahi et al., 2014), but significant differences in the frequency of conventional stroke risk factors were reported in others (Andrew et al., 2014). In the present study, the prevalence of hypertension, dyslipidemia, obesity, current smoking, and alcohol consumption was not significantly different between the two groups, but there was a higher frequency of diabetes in the non-AF group, as reported in the prior study (Lamassa et al., 2001; Mizrahi et al., 2014).

Previous studies have demonstrated that elderly stroke patients were more likely, compared to younger stroke patients, to have poor outcomes after acute ischemic stroke, including increased short-term and long-term mortality rates, dependency rates, and a higher risk of stroke recurrence (Johnston et al., 2002; Kammersgaard et al., 2004; Marini et al., 2004; Rothwell et al., 2005; Palnum et al., 2008; Fonarow et al., 2010; Romero and Wolf, 2013). Elderly acute ischemic stroke patients have poorer outcomes overall (Candelise et al., 1991), these are associated with an enhanced susceptibility to the secondary degeneration in the primary ischemic lesion (Loubinoux et al., 2012). Stroke patients are at highest risk of death in the first weeks after the event, and between 20 and 50% die within the first month depending on type, severity, age, co-morbidity and effectiveness of treatment of complications. Patients who survive may be following with no disability or with mild, moderate or severe disability. However, patients with a history of stroke are at risk of a subsequent event of around 10% in the first year and 5% per year thereafter (Sandu et al., 2015). Moreover, vascular risk factors, including hypertension, obesity, diabetes, dyslipidaemia, hyperhomocystaeinemia, and systemic inflammation increase the probability of silent strokes (Burn et al., 1994).

Findings related to the effects of AF on outcomes in stroke patients have been inconsistent, but most previous studies have reported that AF is associated with higher mortality rates (Candelise et al., 1991; Gustaffson and Britton, 1991; Jørgensen et al., 1996; Lin et al., 1996). A few studies indicated a higher recurrence rate in stroke patients with AF (Sandercock et al., 1992; Lin et al., 1996) and a markedly increased dependency (Karataş et al., 2000). In the present study, we found that there was higher rate of dependency among elderly patients with AF at 3 years after stroke. However, in contrast to previous reports, we found no differences in mortality and recurrence rates between the groups at 1 year and 3 years after stroke. The higher frequency of severe stroke may contribute to the higher rate of dependency among elderly stroke patients with AF, but similar rates of mortality and recurrence among elderly stroke patients across AF groups may be a result of older age (Karataş et al., 2000), and similar frequency of conventional stroke risk factors between the AF group and the non-AF group may partly explain the identical rates of mortality and recurrence among elderly patients (Sandu et al., 2015).

The limitation of this study is that it was a hospital-based study, which might not reflect the general population in China. Furthermore, patients for whom the stroke was fatal, i.e., who died before being hospitalized, were not included in this study. Therefore, there was a relatively low prevalence of AF in this study.

## Conclusion

In this single-hospital-based stroke registry, we compared differences in clinical features, risk factors, and outcomes at 1 year and 3 years post-stroke among elderly stroke patients with and without AF. There was a higher frequency of severe stroke in the AF group than in the non-AF group. While the prevalence of hypertension, dyslipidemia, obesity, current smoking, and alcohol consumption was not obviously different between the two groups, there was a higher frequency of DM in the non-AF group. There was a significantly higher dependency rate at 3 years after stroke in the AF group, but not in mortality rates or recurrence rates across the follow-up periods. These findings suggest that it is crucial to highlight the treatment of elderly stroke patients with AF in order to reduce poor outcomes among the elderly and to reduce the burden of AF in China.

## Author contributions

SW, CZ, and YGZ contributed to the conception and design of the work; YGZ, CZ, CW, YBZ, and SW contributed the data acquisition; YGZ and CZ contributed the analysis and interpretation of data for the work; YGZ contributed to the drafting of the work; and CZ and SW revised the work for important intellectual content. All authors approved of the final version to be submitted and agree to be accountable for all aspects of the work in ensuring that questions related to the accuracy or integrity of any part of the work are appropriately investigated and resolved.

## Funding

This study was supported by Scientific Research Item of Health Department in Heilongjiang Province (contract number: 2011-076) and Youth Science Funds of Heilongjiang Province (contract number: QC2011C130).

### Conflict of interest statement

The authors declare that the research was conducted in the absence of any commercial or financial relationships that could be construed as a potential conflict of interest.

## Abbreviations

AF: atrial fibrillation
BI: Barthel Index
CI: confidence interval
DM: diabetes mellitus
HbA1c: glycosylated hemoglobin
HDL-C: high-density lipoprotein cholesterol
LDL-C: low-density lipoprotein cholesterol
mRS: modified Rankin scale
NIHSS: National Institutes of Health Stroke Scale
OR: odds ratio
TC: total cholesterol
TG: triglycerides.
